# Supplementary material for: The Associations between Perioperative Blood Transfusion and Long-Term Outcomes after Stomach Cancer Surgery
Source: Cancers (Basel). 2021 Oct 29;13(21):5438. doi: 10.3390/cancers13215438 (PMC8582361; doi:10.3390/cancers13215438)

Gastric Cancer

The Associations between Perioperative Blood Transfusion and Long-Term Outcomes after Stomach Cancer Surgery

Fu-Kai Hsu [1,2,†], Wen-Kuei Chang [1,2,†], Kuan-Ju Lin [1,2], Chun-Yu Liu [2,3], Wen-Liang Fang [2,4] and Kuang-Yi Chang [1,2,*]

1 Department of Anesthesiology, Taipei Veterans General Hospital, No. 201, Sec. 2, Shih-Pai Rd, Taipei 112201, Taiwan; kai7234930@gmail.com (F.-K.H.); wkchang@vghtpe.gov.tw (W.-K.C.); atmoonsamo@gmail.com (K.-J.L.)
2 School of Medicine, National Yang Ming Chiao Tung University, Taipei 112304, Taiwan; cyliu3@vghtpe.gov.tw (C.-Y.L.); wlfang@vghtpe.gov.tw (W.-L.F.)
3 Division of Transfusion Medicine, Department of Medicine, Taipei Veterans General Hospital, Taipei 112201, Taiwan
4 Department of Surgery, Taipei Veterans General Hospital, Taipei 112201, Taiwan
* Correspondence: kychang@vghtpe.gov.tw; Tel.: +886-2-28757549; Fax: +886-2-28751597
† These authors contributed equally to this work.

Supplementary Table S1. Frequency table of perioperative packed red blood cell transfusion in surgery for stomach cancer

Unit	Frequency	%	Cumulative %
0	409	71.9	71.9
1	5	0.9	72.8
2	77	13.5	86.3
3	2	0.4	86.6
4	46	8.1	94.7
5	2	0.4	95.1
6	9	1.6	96.7
7	1	0.2	96.8
8	10	1.8	98.6
9	1	0.2	98.8
10	4	0.7	99.5
12	1	0.2	99.6
14	1	0.2	99.8
16	1	0.2	100.0

Supplementary Table S2. Results of logistic regression analysis for inverse probability treatment weighting

	OR	95% CI	*p*
Sex (F vs. M)	0.79	0.43 ~ 1.46	0.456
Body mass index	0.97	0.90 ~ 1.04	0.369
Charlson comorbidity index	1.18	1.00 ~ 1.41	0.056
Preoperative hemoglobin	0.44	0.37 ~ 0.53	< 0.001
Preoperative albumin	0.40	0.20 ~ 0.81	0.011
CEA*	1.08	0.84 ~ 1.39	0.568
CA19-9*	1.07	0.94 ~ 1.22	0.295
Anesthesia time*	1.16	0.62 ~ 2.16	0.648
Blood loss during surgery*	1.10	0.63 ~ 1.92	0.743
Epidural analgesia	1.02	0.41 ~ 2.50	0.970
Previous abdominal surgery	0.65	0.36 ~ 1.20	0.169
Operation date (after 2012 vs. before 2012)	0.78	0.38 ~ 1.60	0.503
Surgery (Subtotal vs. total)	1.29	0.67 ~ 2.49	0.439
Laparoscopic surgery	2.43	1.87 ~ 3.16	< 0.001
Stage			0.868
II vs. I	1.07	0.50 ~ 2.31	0.854
III vs. I	0.88	0.35 ~ 2.20	0.787
Tumor size > 5 cm	0.92	0.51 ~ 1.64	0.767
Histologic differentiation (Poor vs. well to moderate)	1.05	0.59 ~ 1.85	0.867
Lymphovascular invasion	1.20	0.62 ~ 2.34	0.592
Residual tumor on surgical margin	0.12	0.01 ~ 1.53	0.104
Adjunct chemotherapy	1.35	0.68 ~ 2.68	0.397

OR: odds ratio; CI: confidence interval; CEA: carcinoembryonic antigen; CA19-9: carbohydrate antigen 19-9. *On base-2 logarithmic scale

Supplementary Figure S1. Comparisons of absolute standardized differences in the collected variables before and after IPTW

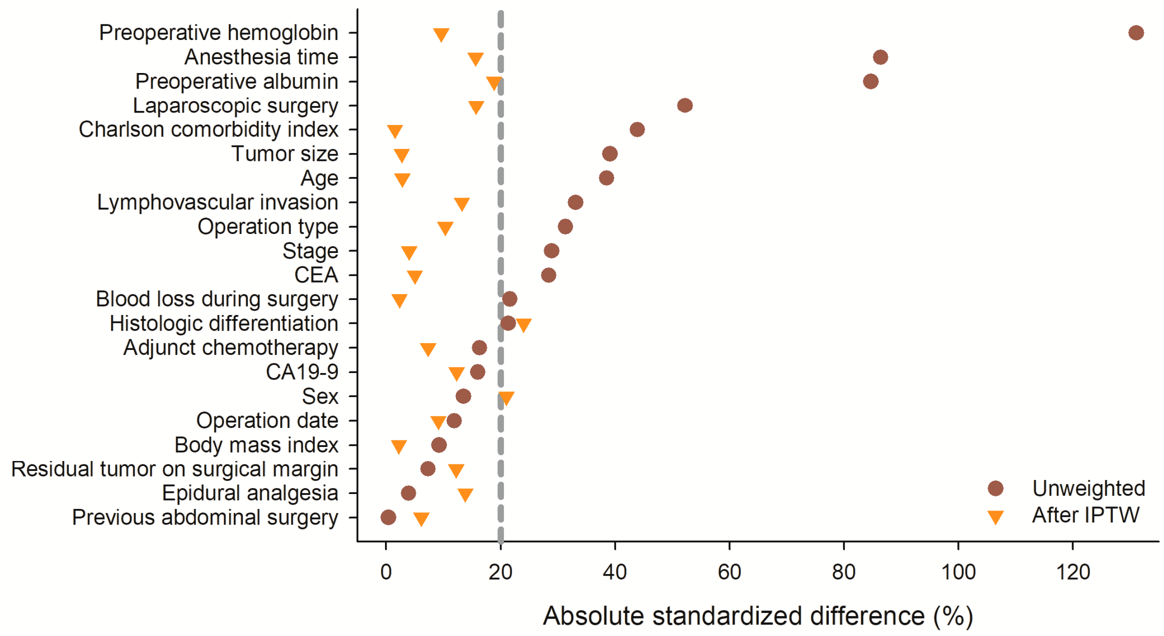

Supplement: Supplementary file 1 [file cancers-13-05438-s001.zip › cancers-1345162-supplementary.pdf]
